# Fluorescent Sensing Platforms for Detecting and Imaging the Biomarkers of Alzheimer’s Disease

**DOI:** 10.3390/bios13050515

**Published:** 2023-04-30

**Authors:** Xingyun Liu, Yibiao Liu, Qiong Liu

**Affiliations:** 1 Department of Chemistry, College of Chemistry and Environmental Engineering, Shenzhen University, Shenzhen 518060, China; liuxingyunfairy20@163.com; 2 Longgang District Central Hospital of Shenzhen, Shenzhen 518116, China; 3 Shenzhen-Hong Kong Institute of Brain Science, Shenzhen Fundamental Research Institutions, Shenzhen 518055, China; 4 Shenzhen Key Laboratory of Marine Biotechnology and Ecology, College of Life Sciences and Oceanography, Shenzhen University, Shenzhen 518055, China

**Keywords:** Alzheimer’s disease, biomarkers, diagnosis

## Abstract

Alzheimer’s disease (AD) is an irreversible neurodegenerative disease with clinical symptoms of memory loss and cognitive impairment. Currently, no effective drug or therapeutic method is available for curing this disease. The major strategy used is to identify and block AD at its initial stage. Thus, early diagnosis is very important for intervention of the disease and assessment of drug efficacy. The gold standards of clinical diagnosis include the measurement of AD biomarkers in cerebrospinal fluid and positron emission tomography imaging of the brain for amyloid-β (Aβ) deposits. However, these methods are difficult to apply to the general screening of a large aging population because of their high cost, radioactivity and inaccessibility. Comparatively, blood sample detection is less invasive and more accessible for the diagnosis of AD. Hence, a variety of assays based on fluorescence analysis, surface-enhanced Raman scattering, electrochemistry, etc., were developed for the detection of AD biomarkers in blood. These methods play significant roles in recognizing asymptomatic AD and predicting the course of the disease. In a clinical setting, the combination of blood biomarker detection with brain imaging may enhance the accuracy of early diagnosis. Fluorescence-sensing techniques can be used not only to detect the levels of biomarkers in blood but also to image biomarkers in the brain in real time due to their low toxicity, high sensitivity and good biocompatibility. In this review, we summarize the newly developed fluorescent sensing platforms and their application in detecting and imaging biomarkers of AD, such as Aβ and tau in the last five years, and discuss their prospects for clinical applications.

## 1. Introduction

Alzheimer’s disease (AD) is a common neurodegenerative disease accompanied by the clinical features of memory loss and cognitive decline [1]. Approximately 34 million people worldwide are suffering from this disease at present. With the rising aging population, more than 131 million people are expected to be affected by this disease by 2050, resulting in a huge burden to individuals and society [1,2]. So far, the pathogenesis of the disease has not been revealed clearly, and there are no effective measures for the treatment in clinical settings. Early diagnosis and intervention are still the only measures to prevent the disease. Amyloid plaques formed by extracellular Aβ peptide precipitation and neurofibrillary tangles (NFTs) formed by intracellular tau protein aggregation are the main pathological features of AD in the brain [3], whose levels in the cerebrospinal fluid (CSF) have been used as the gold standards for neuropathological diagnosis of AD. Alteration in Aβ and tau levels in CSF and plasma may appear many years before symptoms. Tracking these changes in biomarkers is very important to identify asymptomatic AD, and thus, predict the progression of the pathological process.

According to the report by the National Institute on Aging-Alzheimer’s Association (NIA-AA) in 2018 [1,3,4], the quantitative detection of AD biomarkers, including Aβ40 and Aβ42 peptides and phosphorylated and total tau proteins, in CSF was established as the diagnostic criteria for identifying probable AD patients. However, these methods based on CSF biomarkers are not applicable for physical examination of the public due to their invasiveness during sample collection via lumbar puncture. Research showed that biomarkers in CSF can access the blood due to damage to the blood–brain barrier (BBB) in AD [5,6,7,8,9]. In the blood, a low level of Aβ42 and high levels of total and phosphorylated tau proteins indicate damage and degeneration of neurons in the brain [10], and the contents of these biomarkers are altered with the process of AD development. Therefore, detecting readily available blood biomarkers (e.g., Aβ42 peptides, phosphorylated tau proteins and total tau proteins) is becoming a promising strategy for AD diagnosis. In clinical settings, the currently available and practical methods for AD diagnosis are the rating scale tests and brain imaging, including positron emission tomography (PET) [11], single-photon emission computed tomography (SPECT) [12,13,14,15] and magnetic resonance imaging (MRI). However, the rating scale tests are highly dependent on the judgment of doctors, with high subjectivity and inaccuracy. Brain imaging with the above investment is comparably accurate, but the expensive instruments and radioactive tracer for PET block their application in physical examinations and population screening [16,17]. For example, PET detection uses the accumulation of ^11^C or ^18^F isotopes in the metabolism of radionuclides in the human body to reflect the metabolic activities of life. However, the half-lives of ^11^C and ^18^F are relatively short at 20 and 110 min, respectively, and they are quickly metabolized in the body [18].

**Figure 1:**
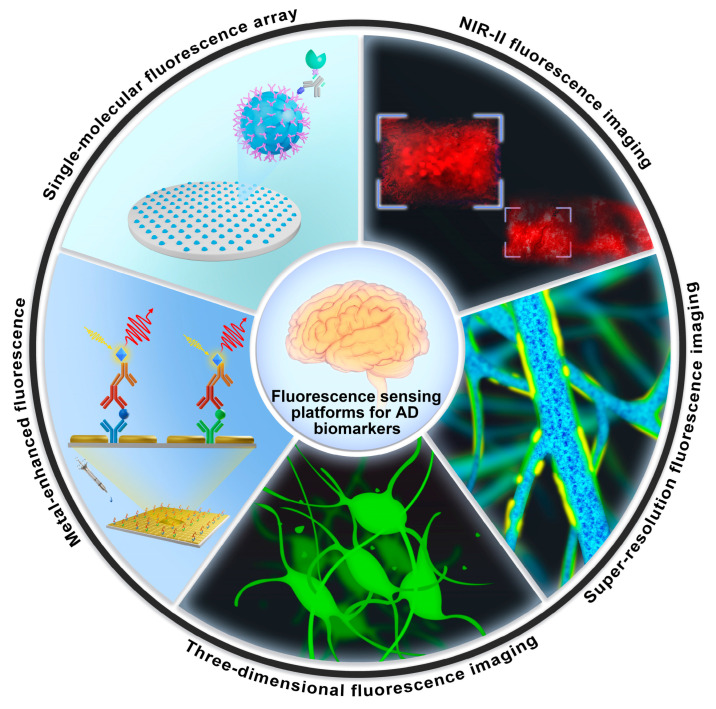
Schematic illustration of enhanced fluorescence-sensing platforms for detecting trace-level AD blood biomarkers and imaging AD features in the brain.

The combination of blood biomarker detection with neuroimaging in the brain could be an effective strategy to enhance the accuracy of early diagnosis. With the rapid progress of sensing techniques, fluorescent methods are used not only to detect the levels of biomarkers in blood but also to instantly image them in the brain based on the low toxicity, high sensitivity and good biocompatibility of fluorescent agents and the excellent sensing performance and accessibility of the technology platform. However, for the detection of a trace amount of samples, such as a drop of blood, the fluorescence signal is weak, and thus, the detection sensitivity needs to be improved, leading to the optical signal detection equipment needing to be much more complex and expensive, which limits its application [19]. At present, a single-molecular array (Simoa) based on the principle of immunofluorescence became a mature technology from Quanterix company that can measure AD blood biomarkers at femtomolar concentrations. As shown in Figure 1, the ultra-high sensitivity of Simoa mainly relies on its femtoliter-sized reaction system to reduce the background noise. This technology requires expensive equipment for the measurement, which is a typical example of using a large-scale detection system to achieve an ultra-sensitive measurement [20]. In contrast to the published articles on the targeting of amyloid aggregates using photoactive probes [21], the targeting of AD using small molecule near-infrared fluorescence probes [22] was discussed. In this review, we excluded the fluorescence methods considering their high cost and restrictions regarding equipment. We focused on the newly developed enhanced fluorescent sensing platforms for their application in detecting AD blood biomarkers, such as Aβ, tau, BACE1 and APOE4. Moreover, the imaging of Aβ plaques and tau tangles in the brain based on fluorescent sensing platforms is also summarized. In brief, this review first overviews some AD biomarkers in the blood from the last five years and then introduces enhanced fluorescence-sensing platforms for detecting them, with further applications in imaging Aβ plaques and NFTs in the brain. The prospects and challenges of those platforms are also discussed in terms of research directions and clinical applications.

## 2. Biomarkers for AD Diagnosis

Compared with cerebrospinal fluid sampling via a lumbar puncture and other invasive methods, using blood as a non-invasive method to detect AD biomarkers has attracted great attention. AD occurs over a long period with various biomarkers, such as Aβ, tau, APOE, BACE1 and microRNAs, showing up in the blood. The main pathological features of AD are senile plaques, which are mainly formed by the aggregation of Aβ and neurofibrillary tangles (NFTs) formed by the hyperphosphorylation of tau (P-tau), with both of these leading to the death of neurons [23,24]. Therefore, Aβ (including Aβ monomers, oligomers and plaques) [17,25,26] and tau (including total tau proteins and phosphorylated tau proteins) [9,20,27] are generally regarded as the two most important types of AD biomarkers in blood. In addition, other blood biomarkers were also reported, such as the specific microRNAs [28], BACE1 [29,30] APOE [31,32,33,34] and metal ions [35,36].

Many studies showed that the level of Aβ protein begins to change 10–15 years before clinical manifestation [25]. As the major component of senile plaques, Aβ is derived from amyloid precursor protein (APP). APP metabolism is mediated by three hydrolases, i.e., α-, β- and γ-secretases. Aβ is produced by APP cleavage sequentially through β- and γ-secretases. Polypeptides Aβ40 and Aβ42 containing 40 and 42 amino acids, respectively, are the two most important isoforms [26]. Normally, the production and clearance of Aβ peptides should be balanced, but in AD patients, increased production and decreased clearance of Aβ lead to abnormal extracellular accumulation of Aβ40 and Aβ42 peptides. Since the concentration of Aβ40 in CFS is ten times that of Aβ42, the concentration of Aβ40 does not change significantly during the process of AD, and the ratio of Aβ42/Aβ40 is more accurate than that of a single indicator [37]. The abnormally aggregated Aβ42 monomer self-assembles into soluble Aβ oligomers, which then form fibrils and plaques that eventually lead to tau pathology and subsequent neuronal dysfunction [38]. Soluble Aβ oligomers are the main toxic substances and are more specifically involved in neuronal death and cognitive impairment than insoluble fibrils [38,39].

Tau proteins play physiological roles in stabilizing microtubules, reducing the dissociation of tubulin molecules and inducing microtubules to form bundles. There are six isoforms of tau proteins containing 352, 381, 383, 410, 412 and 441 amino acids [40]. In the case of full-length tau, which contains 441 amino acids, there are a total of 85 possible phosphorylation sites that regulate the binding of tau to microtubules to keep them stable [41]. In healthy people, a tau protein contains 2–3 phosphate groups, while in AD patients, tau is abnormally hyperphosphorylated and can contain 5–9 phosphate groups per protein. Hyperphosphorylation of tau reduces the binding affinity between tau and microtubules and grabs other microtubule-associated proteins (MAPs) from microtubules, which leads to the formation of NFTs and ultimately results in microtubule depolymerization and impaired neuronal function [42].

In addition, APOE, BACE1 and some microRNAs are used to analyze the pathology of AD. Apolipoprotein E (APOE) is a major lipid transporter in the brain, with three main isoforms (APOE2, APOE3 and APOE4) [31]. The APOE4 allele is one of the most important genetic risk factors [43]. APOE4 heterozygous carriers have a 2–3 times higher risk of AD than noncarriers, while homozygous individuals have a 12 times higher risk of AD than noncarriers [31,44]. APOE2 carriers have a protective effect compared with APOE3 and APOE4 carriers [45]. Although the exact mechanism of how APOE4 mediates AD pathology is still unclear, APOE4 was accepted in clinical settings as a biomarker to distinguish AD candidates and healthy controls. BACE1 (i.e., β-secretase) is an aspartic acid protease that mediates the cleavage of APP to release Aβ. Therefore, the reduction in BACE1 can effectively reduce the overproduction of Aβ [46]. Studies showed that BACE1 inhibitors effectively reduce excessive production and abnormal accumulation of Aβ [47]. MicroRNAs are small endogenous noncoding RNAs [48]. Some microRNAs are regulatory factors used to regulate the expression of APP and the production and clearance of Aβ during their formation. MicroRNAs are very stable in blood, plasma and CSF, and are gradually becoming biomarkers for the detection of AD [49]. For example, Arun Richard Chandrasekaran et al. [50] developed AD-related miR-107 native assays, achieving ultrasensitive detection of miR-107 without amplification or labeling. The metabolism of human cells produces reactive nitrogen species (RNS) and reactive oxygen species (ROS). ROS has a dual role in the body. Overactivity of ROS will play a destructive role in accelerating human aging and cell damage [51]. The content of ROS in AD patients is higher than that in normal people. ONOO^−^ is a combination product of superoxide and nitric oxide, which is an active substance that causes great harm via oxidative stress. Wang et al. [52] designed a peroxynitrite (ONOO^−^)-activated near-infrared probe, namely, Rd-DPA3, whose detection limit of ONOO^−^ was 3.4 nM, which is higher than that of other ROS. In this study, the structure and lipophilicity of the probe scaffold were adjusted to improve the ability of the current probe to cross the weak blood–brain barrier and realize the monitoring of ONOO^−^ in vivo.

## 3. Enhanced Fluorescence-Sensing Platforms for the Detection of AD Blood Biomarkers

As mentioned above, AD blood biomarkers are important for early diagnosis, predicting the stage of disease and monitoring drug effects [9,10]. Compared with CSF sampling using a lumbar puncture and other invasive methods, blood detection has attracted great attention. However, blood-analysis methods also have certain limitations, such as very low levels of pathological proteins transferring from the brain to the blood and a wide range of interfering proteins. Therefore, there is an urgent need for ultrasensitive, stable and repeatable methods to detect AD biomarkers in blood [53]. The fluorescence detection technique was previously tried for detecting AD blood biomarkers, which exhibited some advantages, including high sensitivity, simple operation and fast detection speed compared with traditional protein analytical methods. However, the fluorescence signal is very weak when the concentration of the target analyte is low. To obtain higher sensitivity, there are generally two strategies. One is to use large detection equipment to identify weak signals, and the other is to amplify weak fluorescence signals. Under physiological conditions, the concentration of AD blood biomarkers is only in the order of pg/mL, which is beyond the detection limit of ordinary fluorescence detection methods [19]. Considering the complexity and high cost of large-scale instruments, here we mainly summarize the application and advance of enhanced fluorescence-sensing platforms in the detection of AD biomarkers in blood, including Aβ, tau, metal ions and microRNA.

### 3.1. Amyloid-β Peptide

**Figure 2:**
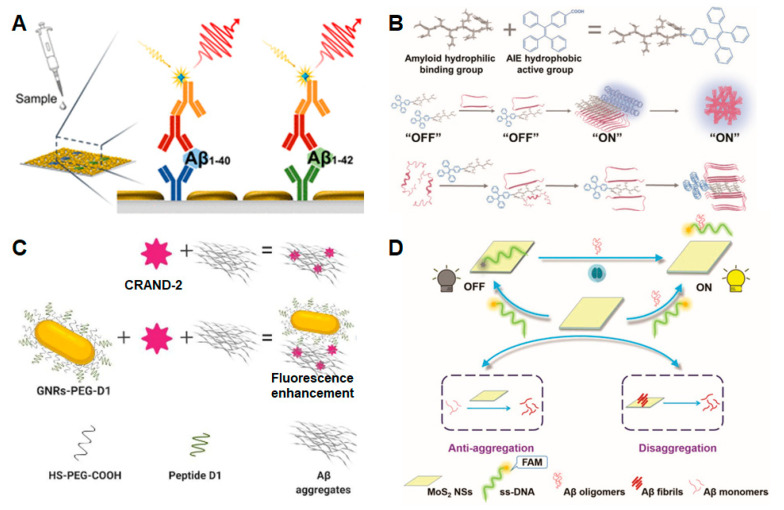
Schematic diagrams of four enhanced fluorescence-sensing platforms. (**A**) Enhanced fluorescence-sensing platform based on plasmonic gold nanoislands for the detection of Aβ40 and Aβ42. Copyright (2021) ACS publications [54]. (**B**) Design of an amyloid-AIE fluorescence molecule and the principle of detecting amyloid proteins. Copyright (2022) Wiley [55]. (**C**) Enhanced fluorescence sensor based on gold nanorods for Aβ aggregates. Copyright (2020) MDPI [56]. (**D**) Fluorescence-sensing platform based on MoS_2_ nanosheets for monitoring Aβ oligomers. Copyright (2020) RSC publications [57].

The Aβ peptide is a classical biomarker of AD in its early stage. It includes multiple forms, such as Aβ40, Aβ42, Aβ oligomers and Aβ fibers. To improve the sensitivity of fluorescence detection, Chen et al. [54] constructed an enhanced fluorescence platform for the ultrasensitive detection of Aβ40 and Aβ42 based on gold nanoislands. The research procedures included the screening of Aβ antibody, optimizing the conditions and adjusting the program to realize near-infrared fluorescence enhancement of a plasmonic gold nanochip. The detection limit of this method reached 0.1 pg/mL. As shown in Figure 2A, the plasmonic gold nanochip surface contains a gold nanoisland layer with nonuniform gap separation. The gold nanoislands provide a powerful surface plasmon resonance enhancement, which, in turn, significantly increases the fluorescence signal.

Tang et al. [55] conjugated a hydrophilic peptide GNNQQNY (G7) with the hydrophobic AIE fluorescent molecule triphyenylvinyl benzoic acid (TBA) to form a G7-TBA molecule for the multifunctional detection and regulation of Aβ (Figure 2B). As an Aβ probe G7-TBA solves the problems of poor water solubility and low permeability of traditional AIE molecules. The hydrophilic peptide of G7, i.e., GNNQQNY, is a short fragment of the yeast prion protein Sup35 with a binding motif to interact with Aβ and human islet amyloid polypeptide (hIAPP) in type II diabetes. According to the AIE luminescence mechanism, the G7-TBA probe has low fluorescence in an Aβ monomer state and high fluorescence in an aggregated state. The G7-TBA probe showed a higher affinity for amyloid aggregates than the commercial ThT and amyloid aggregates.

Francisco Morales-Zavala et al. [56] combined CRANAD-2 with functionalized gold nanorods (GNRs) to significantly improve the fluorescence signals of CRANAD-2 for Aβ aggregates (see Figure 2C). The detection method consists of two parts: CRANAD-2 fluorescent probe and GNRs. The CRANAD-2 probe is a curcumin analog that has a high affinity for insoluble amyloid aggregates, such as Aβ fibrils (*K_d_* = 38 nM); however, its low quantum yield (20%) makes the probe difficult to detect [58], and this limited the application of the probe in detecting amyloid aggregates in AD. The surface of the gold nanorods was modified with HS-PEG-OMe and HS-PEG-COOH, and then coupled with a peptide with selective recognition of Aβ aggregates (please refer to the author’s article) [59]. The functionalized GNRs enhance the fluorescence signal of the CRANAD-2 probe and target Aβ aggregates that are impossible to be detected using CRANAD-2 alone. This method can be used for the detection of amyloid in vivo, and it provides a more reliable way for the fluorescence imaging of AD pathology.

Lingna Kong et al. [57] combined fluorescent dye-labeled single-stranded DNA (ssDNA) with molybdenum disulfide nanosheets (MoS_2_ NSs) and realized the detection of Aβ oligomers with a detection limit of 3.1 nM (Figure 2D). The fluorescence group FAM, which specifically recognizes Aβ oligomers, was labeled on single-stranded DNA and then coupled with MoS_2_ NSs. At this time, fluorescence quenching is caused by the action of van der Waals forces. After the addition of an Aβ oligomer, a hybrid structure was formed between the Aβ oligomer and ssDNA, resulting in the stripping of fluorescent dye-labeled single-stranded DNA from the molybdenum disulfide nanosheet and fluorescence recovery. By adding different concentration gradients of Aβ oligomers and measuring different fluorescence signals, the ultra-low concentration of Aβ oligomers was detected. In addition, the team also found that MoS_2_ NSs can inhibit Aβ aggregation and degrade the formed Aβ fibrils.

### 3.2. Tau

Tau protein plays an important role in stabilizing microtubules; however, its hyperphosphorylation destabilizes microtubules and leads to the pathogenesis of AD. Total tau and phosphorylated tau in CSF have been widely accepted in clinical settings as the key biomarkers of AD. With the advances in analytical techniques, the phosphorylated tau at amino acid positions 181 (p-tau181) and 217 (p-tau217) are also considered the biomarkers of AD in blood.

**Figure 3:**
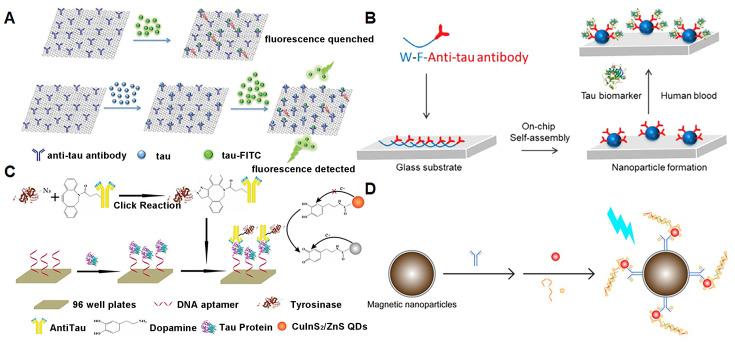
Fluorescence-sensing platforms for the detection of tau proteins. (**A**) Fluorescence quenching based on graphene oxide for detecting the tau protein. Copyright (2018) RSC publications [60]. (**B**) Anti-tau arrays on chip for detecting tau protein in the blood. Copyright (2021) Elsevier [61]. (**C**) The fluorescence immunoassay based on CuInS_2_/ZnS quantum dots for the detection of tau protein. Copyright (2019) Springer [62]. (**D**) Fluorescence-sensing platform based on magnetic nanoparticles for detecting AD biomarkers. Copyright (2019) Ivyspring International Publisher [63].

Yao et al. [60] developed a non-enzyme-linked secondary antibody to recognize proteins and an enzyme substrate to generate optical signals, which greatly reduced the operation steps for tau detection and improved the experimental accuracy (Figure 3A). The team adsorbed anti-human tau antibodies onto graphene oxide (GO) surfaces to provide specific binding sites for tau. The tau protein, which binds to the binding site, is added to a buffer containing antibody-conjugated GO, followed by the addition of standard fluorescein isothiocyanate-labeled tau (tau-FITC). The GO surface is both a nanoscale binding platform and an energy acceptor, which quenches the fluorescence of tau-FITC that is close to and adsorbed to the graphene surface, while those that are not close to the graphene surface emit fluorescence. The number of binding sites for modified GO is limited, where the more tau protein that is adsorbed, the less tau-FITC that is adsorbed and the stronger the fluorescence signal generated by free tau-FITC in the solution. Therefore, the fluorescence intensity can be controlled by the tau protein concentration, and the tau protein can be detected according to the changes before and after fluorescence.

Sun et al. [61] developed fluorescent peptide nanoparticle (f-PNPs) arrays to simultaneously detect multiple signals of AD blood biomarkers (Figure 3B). Tryptophane-phenylalanine (WF) dipeptide is an effective self-assembly sequence that induces the formation of f-PNPs arrays on glass substrates. The f-PNPs arrays are modified by anti-tau antibodies to target serum tau protein. This method not only shows a high signal-to-noise ratio for AD diagnosis but also detects multiple signals simultaneously, such as tau protein concentration and aggregation stages.

Zhang et al. [62] established a redox-mediated fluorescent immunoassay for the detection of tau protein based on dopamine (DA)-functionalized CuInS_2_/ZnS quantum dots (Figure 3C). DA is modified on the surface of CuInS_2_/ZnS quantum dots via amide reactions. A biotin-modified aptamer, tau protein and TRY-anti-tau were sequentially added to the 96-hole polystyrene plate coated with avidin. Tyrosinase (TYR) is an oxidase that catalyzes dopamine oxidation. After washing off the excess TRY-anti-tau, CuInS_2_/ZnS-DA quantum dots were added. Under the catalysis of TYR, the DA in CuInS_2_/ZnS-DA is partially oxidized to dopamino quinone, which can be used as a quenching agent of CuInS_2_/ZnS quantum dots, triggering fluorescence quenching. By modifying TYR, the sensitivity of the fluorescence-sensing platform was improved to a detection limit of 9.3 pM and a linear range of 10 pM~200 nM.

Chan et al. [63] developed an ultrasensitive multiple assay using a hybrid antibody-aptamer immunoassay, and then amplified the target signal via DNA amplification technology, with a sensitivity down to the femtomolar level (Figure 3D). For example, the simultaneous detection and recognition of p-tau181 and tau441 can be achieved using two different morphometric magnetic probes with detection limits of 3.6 fM and 4.3 fM, respectively. This method uses fewer samples, does not require pre-labeled antibodies and can distinguish protein isomerism.

### 3.3. Other Biomarkers in the Blood

In addition to the most important Aβ and tau biomarkers, there are some other AD biomarkers in the blood, such as microRNA, metal ions, APOE and BACE1. We also summarize the fluorescence-sensing platform for detecting these AD blood biomarkers.

**Figure 4:**
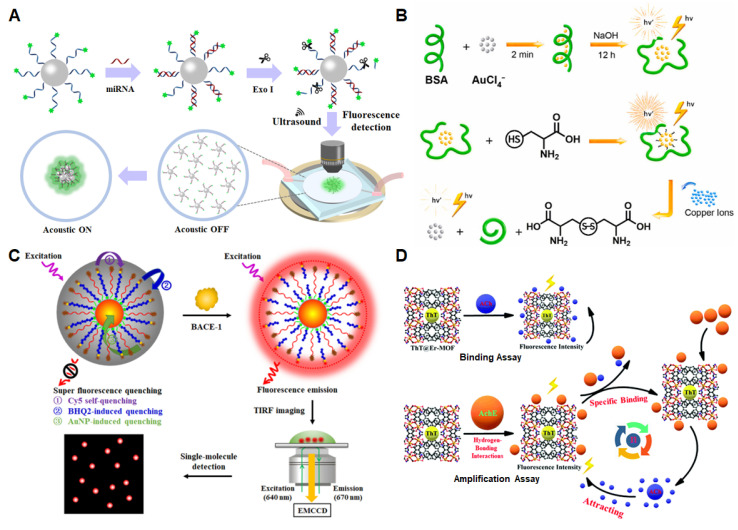
Fluorescence-sensing platforms for the detection of microRNA, metal ions and pathological enzymes of AD. (**A**) Fluorescence enhancement based on acoustic aggregation for detecting miRNA. Copyright (2021) Elsevier [64]. (**B**) Fluorescence switch for the detection of Cu^2+^. Copyright (2021) Elsevier [35]. (**C**) Fluorescence nanosensors based on the self-assembly of superquenched AuNPs for the detection of BACE-1. Copyright (2021) ACS publications [66]. (**D**) Fluorescence-sensing platform based on ThT@Er-MOF for AChE detection. Copyright (2020) RSC publications [67].

Specific miRNAs in the blood are biomarkers for the early diagnosis of AD, and the detection procedure is minimally invasive and painless. Zhang et al. [64] developed a method that combines acoustic-aggregation-induced particles with fluorescence enhancement, providing a new strategy for the detection of the AD biomarker miRNA (Figure 4A). Briefly, carboxyfluorescein (FAM)-modified DNA probes were coupled with carboxyl-functionalized polystyrene particles. The DNA probes on fluorescein-labeled particles were complementary to the miRNAs and base-paired with them to form double-stranded structures. DNA exonuclease was then introduced to cut the unhybridized DNA probe to release FAM, and the double-stranded structure with bases complementary to the miRNA was protected. When the ultrasound is turned off, the particles modified by the FAM-labeled DNA probe are in a dispersed state, and the fluorescence signal is weak. However, when the ultrasound is turned on, driven by sound waves with a frequency of 530 kHz and a voltage of 500 mV, the particles modified by the FAM-labeled DNA probes will move and gather in the center of the chip, and strong fluorescence can be detected at this time. The research group verified miRNA-101 with a detection limit of 5 fM.

Studies showed that Cu^2+^ is involved in the regulation of Aβ fibril assembly and neurotoxicity. Cysteine plays the role of redox signaling in AD, and thus, the detection of Cu^2+^ and cysteine is essential for the study of the pathology of AD [65]. In 2021, Niu et al. [32] developed a label-free fluorescent probe to continuously detect the levels of cysteine and Cu^2+^. As shown in Figure 4B, under alkaline conditions, gold nanoclusters (AuNCs) were prepared using the reaction of HAuCl_4_ with bovine serum protein (BSA-AuNCs). The electron transition of BSA-AuNCs molecular orbitals produced fluorescence signals. When cysteine was added, it filled the gap on the BSA-AuNCs surface and reacted with BSA-AuNCs, resulting in a significantly enhanced fluorescence signal. With the addition of Cu^2+^, cysteine is oxidized and the Au-S bond between cysteine and BSA-AuNCs is destroyed. Cu^2+^ will also interact with tryptophan in BSA, which will destroy the stability of BSA-AuNCs and lead to fluorescence quenching. The detection limit of Cu^2+^ in AD mice was 0.1465 μM.

Zhang et al. [66] constructed a self-assembly superhardened gold nanoparticle (AuNP) nanosensor to detect and image BACE-1 instantaneously in living cells (Figure 4C). The nanoprobes consist of three parts: multiple fluorophore-labeled peptide probes, an assistant DNA containing a black hole quencher 2 (BHQ2) and AuNPs. The fluorescein-labeled peptide probes and BHQ2-labeled DNA probes assist with self-assembly on the surface of AuNPs, resulting in superfluorescence quenching. When BACE1 is added, this enzyme catalyzed the cleavage of the fluorophore-labeled peptide probes to release abundant fluorophores. The fluorophores signal can be displayed directly using single-molecular imaging. This enhanced fluorescent sensor has the advantages of high efficiency, low detection limit and good stability. The quenching efficiency of the self-assembly process is up to 98.37% and the background fluorescence is close to zero. Combined with the single molecule detection technique, the sensor can sensitively detect BACE1 with a detection limit of 26.48 pM. This nanoprobe can also be used to instantaneously image endogenous BACE1 in living cells.

Ding et al. [67] introduced the fluorescent dye thioflavin (ThT) into the three-dimensional material Er-MOF (a novel metal–organic framework [Er(L)(DMF)_1.27_]_n_) to construct a ratiometric fluorescence sensor based on ThT@Er-MOF. The ThT@Er-MOF sensor successfully achieved ultrasensitive detection of AD biomarkers, such as presenilin 1 (PS1), Aβ and acetylcholine (ACh), with three different detection strategies. Mutation in the PS1 gene leads to the pathology of AD [68]. ACh is a neurotransmitter that can be degraded by acetylcholinesterase (AchE), which is considered one of the biomarkers of AD [69]. Figure 4D introduces two different ACh detection processes based on the ThT@Er-MOF-modified fluorescence-sensing platform.

There were many valuable studies on enhanced fluorescence-sensing platforms for detecting AD blood biomarkers. Detailed information about each biomarker and the analytical performance, including the limit of detection (LOD) is shown in Table 1.

## 4. Fluorescence-Sensing Platforms for Imaging AD Biomarkers in the Brain

The detection of AD biomarkers in blood has the advantages of less invasiveness, lower cost and faster response time. However, currently, its accuracy is not good enough for clinical diagnosis. The combination of blood biomarkers’ detection with brain imaging analysis can greatly improve the accuracy of diagnosis. Enhanced fluorescence-sensing techniques also show important value in brain imaging in addition to their advantage in detecting trace amounts of AD biomarkers in blood. This section mainly reviews the application of fluorescence-sensing technology in high-resolution imaging of AD biomarkers in the brain.

**Figure 5:**
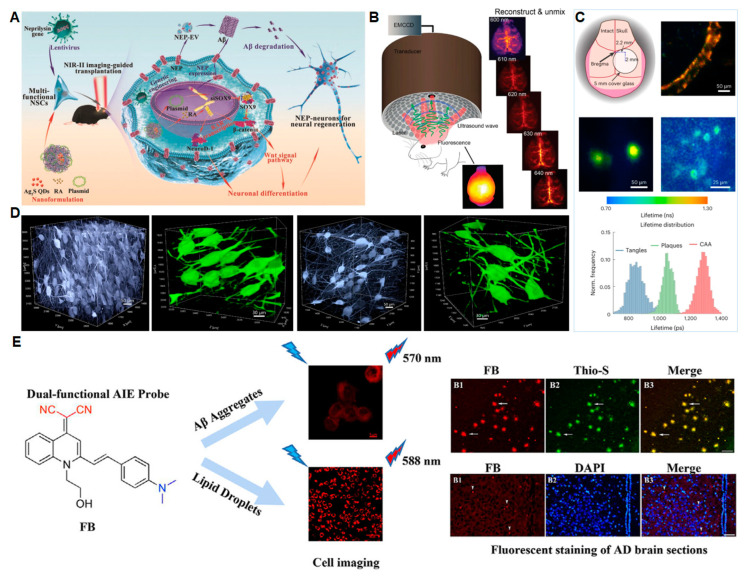
Fluorescence-sensing platforms for imaging AD biomarkers in the brain. (**A**) The schematic of a multifunctional PPAR-SiSOX9 nanoformulation for the treatment and imaging of AD. Copyright (2021) Wiley [81]. (**B**) The hybrid epifluorescence–vMSOT system for tau mapping. Copyright (2022) Springer [82]. (**C**) Intact skull imaging for Aβ plaques and NFTs. Copyright (2023) Springer Nature [83]. (**D**) High-resolution 3D crops of NA neurons according to the spatiotemporal three-dimensional imaging. Copyright (2022) Springer [84]. (**E**) Dual-functional AIE probe for imaging Aβ aggregates and lipid droplets (FB: a quinoline derivative). Copyright (2020) Elsevier [85].

Real-time imaging plays an important role in tracing the distribution and alteration of neuronal development and disease processes, as well as the efficiency of drug treatment in living animals. Wang et al. [81] developed a PBAE-PLGA-Ag_2_S-RA-SiSOX9 (PPAR-SiSOX9) (PBAE: poly(beta- amino esters), PLGA: poly(D,L-lactide-co-glycolide)) nanoformulation with high gene/drug deliverability to neural stem cells (NSCs). After co-transfection with the lentivirus-carrying neprilysin (NEP) gene, the multifunctional NSCs were then transplanted into mouse brains with the guidance of second near-infrared (NIR-II) imaging. The therapeutic potential of NSCs that clear Aβ plaques and regenerate nerve cells through NEP expression were investigated following the treatment. For accurate stereotactic transplantation, fluorescence imaging based on Ag_2_S quantum dots (QDs) was used in real time. As shown in Figure 5A, the NIR-II imaging based on Ag_2_S QDs can be used to monitor the whole process.

High-resolution imaging of whole-brain scale tau deposits is of great significance. Patrick et al. developed a non-invasive tau-targeted probe and imaged tau deposits in the whole mouse brain using a multi-spectral optoacoustic tomography (vMSOT) system, fluorescence imaging setup and data analysis [83]. Figure 5B shows the hybrid fluorescent–vMSOT system that was established for tau mapping across the entire mouse brain. This high-resolution imaging platform is of great significance for studying tau spreading and clearance and evaluating the therapeutic effect of tau-targeting drugs.

Hak Soo Choi et al. [83] reported a NIR heptamethine fluorophore ZW800-1C with a peak excitation of 753 nm and emission of 772 nm in the NIR window. This NIR fluorescent probe can combine with Aβ and tau simultaneously and can be used as a multifunctional probe for AD imaging in vivo. The probe can be applied to image the intact skull. As shown in Figure 5C, both plaques and NFTs can be imaged using the intact skull. In addition, the NIR fluorescent probe can cross the BBB, which is very important for imaging encephalopathy. Abris Gilvesy et al. studied the spatiotemporal characterization of the process of cellular tau pathology in the human locus coeruleus using 3D imaging. This study addressed the relationship between the locus coeruleus cell structure in the 3D view and tau cytoskeletal pathology and the possible transmission mode of disease-associated tau (Figure 5D) [84].

Yan et al. [86] designed two quinoline-based AIE probes to perform high-resolution imaging of Aβ plaques and lipid droplets at the cellular level and in the brain (Figure 5E). It would be helpful to specify what the probe is enhancing fluorescence of, such as “the Aβ plaques”. To target lipid droplets, the research team introduced a fat-soluble substance to the probe, which facilitates its penetration of the blood–brain barrier. The detection limit reached 26.9 nM. With further development, this high-resolution imaging probe has great promise for AD clinical diagnosis.

## 5. Conclusions and Prospects

Alzheimer’s disease poses a serious threat to cognitive function and quality of life in affected individuals. Its pathogenic factors are very complex. Aβ aggregation, tau hyperphosphorylation, neuroinflammation and even intestine flora change were all reported to be associated with AD; however, its pathogenesis remains unclear [1,86,87]. Therefore, early detection and intervention are still the only measures to prevent this disease. The enhanced fluorescence-sensing platform is a technique to improve the sensitivity of fluorescence detection through the plasma resonance effect on the basis of classical fluorescence analysis. A large number of studies showed that trace amounts of AD biomarkers in blood can be detected well by fluorescence enhancement technology, such as Aβ40, Aβ42, T-tau and P-tau. Furthermore, fluorescence-sensing platforms also provide great potential in high-resolution, three-dimensional and living biological imaging. A combination of blood biomarkers detection and brain imaging can significantly improve the accuracy of distinguishing AD patients at different stages, including subjective cognitive decline (SCD), mild cognitive impairment (MCI) and dementia. As an easy method and readily available instrument, fluorescence enhancement platforms provide a promising future in the application of AD diagnosis, mainly including the following three aspects:

(1) Application of novel composite nanomaterials. The accuracy and sensitivity of the analytical method can be significantly improved via the development of special structures and biocompatible nanomaterials combined with fluorescence enhancement technology.
(2) In-depth application of near-infrared fluorescence probes. The biggest defect of fluorescence detection is the decrease in resolution with the increase in fluorescence emission depth, and thus, only the outer surface of the brain can be identified. Furthermore, the fluorescence wavelength is below 550 nm, and some biological substances themselves also fluoresce in this range, which interferes with the detection of organisms in vivo [12,13,15]. With the advent of near-infrared fluorescence imaging, the above problems are gradually solved. The wavelength range of NIR fluorescence imaging is between 700 nm and 2500 nm, which greatly enhances the penetration depth and reduces the interference of biomaterials themselves [88].
(3) Simultaneous detection of different forms of aggregates. For example, Aβ has many aggregation forms, and the current method still utilizes the single detection of only one form. Simultaneous detection of different aggregation forms can not only reduce the cost but also save time and samples, leading to the method being simple, fast and sensitive for the trace analysis of AD biomarkers.

In summary, we believe enhanced fluorescence techniques may be the most promising diagnostic technology for detecting AD blood biomarkers. Although the accuracy of diagnosing AD based on blood biomarkers still needs to be improved, it is the least harmful diagnostic method for patients. Currently, fluorescent detection is still limited to being used by professionals. An integrated, miniaturized testing equipment may be the ultimate layman’s product. As the aging population grows, non-invasive enhanced fluorescence techniques for both blood detection and brain imaging provide great potential for the early diagnosis of AD in clinical settings.

## Figures and Tables

**Table 1:** Fluorescence-sensing platforms based on nanomaterials for detecting AD blood biomarkers.

Nanomaterials	Biomarkers	LOD	Clinical Samples	References
Cu-BTC/Tb	Aβ40	0.3 nM	Human blood	[70]
3D hydrogel	Aβ	0.5 pM	Human serum	[71]
pGOLD	Aβ	0.1 pg/mL	Human blood	[54]
H-USM/BHQ-1	Aβ oligomers	28.4 pg/mL	/	[72]
AuNP-RAMRA	Aβ oligomers	22.3 pM	AD mice	[73]
ThT@Er-MOF	AchE	0.03226 nM	/	[67]
L-MOF	Aβ oligomers	0.4 pg/mL	Human serum	[74]
LMOF/Apt-Au	Aβ oligomers	0.3 pM	Human serum	[75]
CDs@Eu/GMP	Aβ	0.17 nM	Brain tissue of rat	[76]
MoS_2_ NSs	Aβ oligomers	3.1 nM	Brain tissue of AD mice	[57]
FAM-AptAβ@PBNPs	Aβ oligomers	1.0 nM	Human CSF	[77]
CuInS_2_/ZnS quantum dots	Tau protein	9.3 pM	Human serum	[62]
GO	Tau protein	0.14 nM	Human samples	[60]
WS_2_ nanosheet	BACE1	66 pM	Rat CSF	[46]
GQD-CM	APOE4	18.6 pg/mL	Human plasma	[78]
AuNPs	BACE-1	26.48 pM	/	[66]
PS nanoparticle	miRNA-101	5 fM	Human serum	[64]
GOX-SYBR	miRNA-137, miRNA-142	82 nM	Human serum	[79]
WS_2_ nanosheets	miR-29a	745 pM	Human serum	[80]
BSA-AuNCs	Cu^2+^	0.1465 μM	Mice sample	[35]

## Data Availability

Not applicable.
